# Dynamic ^18^F-FDG PET imaging of liver lesions: evaluation of a two-tissue compartment model with dual blood input function

**DOI:** 10.1186/s12880-021-00623-2

**Published:** 2021-05-25

**Authors:** Jingnan Wang, Yunwen Shao, Bowei Liu, Xuezhu Wang, Barbara Katharina Geist, Xiang Li, Fang Li, Haitao Zhao, Marcus Hacker, Haiyan Ding, Hui Zhang, Li Huo

**Affiliations:** 1 Department of Nuclear Medicine, Chinese Academy of Medical Sciences and Peking Union Medical College Hospital, Beijing, People’s Republic of China; 2 Department of Biomedical Engineering, Tsinghua University, Beijing, People’s Republic of China; 3 Division of Nuclear Medicine, Department of Biomedical Imaging and Image-Guided Therapy, Medical University of Vienna, Vienna, Austria; 4 Department of Liver Surgery, Chinese Academy of Medical Sciences and Peking Union Medical College Hospital, Beijing, People’s Republic of China; 5 Beijing Key Laboratory of Molecular Targeted Diagnosis and Therapy in Nuclear Medicine, Chinese Academy of Medical Sciences and Peking Union Medical College Hospital, Beijing, People’s Republic of China

**Keywords:** FDG, Kinetic model, Dual input function, Hepatocellular carcinoma, Intrahepatic cholangiocarcinoma

## Abstract

**Background:**

Dynamic PET with kinetic modeling was reported to be potentially helpful in the assessment of hepatic malignancy. In this study, a kinetic modeling analysis was performed on hepatocellular carcinoma (HCC) and intrahepatic cholangiocarcinoma (ICC) from dynamic FDG positron emission tomography/computer tomography (PET/CT) scans.

**Methods:**

A reversible two-tissue compartment model with dual blood input function, which takes into consideration the blood supply from both hepatic artery and portal vein, was used for accurate kinetic modeling of liver dynamic ^18^F-FDG PET imaging. The blood input functions were directly measured as the mean values over the VOIs on descending aorta and portal vein respectively. And the contribution of hepatic artery to the blood input function was optimization-derived in the process of model fitting. The kinetic model was evaluated using dynamic PET data acquired on 24 patients with identified hepatobiliary malignancy. 38 HCC or ICC identified lesions and 24 healthy liver regions were analyzed.

**Results:**

Results showed significant differences in kinetic parameters ${K}_{1}-{k}_{4}$, blood supplying fraction ${f}_{A}$, and metabolic rate constant ${K}_{i}$ between malignant lesions and healthy liver tissue. And significant differences were also observed in ${K}_{1}$, ${k}_{3}$, ${f}_{A}$ and ${K}_{i}$ between HCC and ICC lesions. Further investigations of the effect of SUV measurements on the derived kinetic parameters were conducted. And results showed comparable effectiveness of the kinetic modeling using either SUVmean or SUVmax measurements.

**Conclusions:**

Dynamic 18F-FDG PET imaging with optimization-derived hepatic artery blood supply fraction dual-blood input function kinetic modeling can effectively distinguish malignant lesions from healthy liver tissue, as well as HCC and ICC lesions.

## Background

Hepatobiliary malignancy including hepatocellular carcinoma (HCC) and intrahepatic cholangiocarcinoma (ICC) accounts for over 90% of all primary liver malignancy, which is the fifth leading cause of cancer related deaths [1]. Distinguishing malignant from benign lesions, as well as HCC from ICC, still remains clinically challenging despite traditional imaging modalities have been utilized for the assessment of the disease [2]. ^18^F-fluorodeoxyglucose positron emission tomography (^18^F-FDG PET) is a reliable functional imaging tool that provides valuable information for staging, predicting prognosis and evaluating therapeutic response, albeit with relatively limited sensitivity in detecting well-differentiated tumors [3]. As a glucose analog, the uptake of ^18^F-FDG is a biological process of glucose consumption at cellular levels through intra-cellular transportation and phosphorylation. Tracking the dynamics of ^18^F-FDG in vivo has the potential to better understand the different glycolytic characteristics between normal and tumors cells. Dynamic PET with kinetic modeling provides an overall view of tracer behavior and quantitative kinetic parameters can be derived to characterize the perfusion and metabolism process [4]. High correlations between the glycolytic enzyme activities and kinetic parameters were reported in previous studies. These kinetic parameters have the potential to serve as an important complement to the commonly used standard uptake values (SUVs) measured in static PET imaging [5, 6]. Previous studies have reported that dynamic PET with kinetic modeling could be used for differential diagnosing, pathological grading and therapeutic evaluating in hepatic malignancy [7, 8].

The blood input function is essential for quantitative analysis in kinetic modeling of dynamic PET [4]. Earlier studies used a single-blood input function (SBIF) from the hepatic artery (HA) for kinetic modeling in the liver [5, 6],and it was recently shown that FDG kinetics in HCC and healthy liver regions could also be modeled solely by using a single blood input function from the portal vein (PV) [9]. Since the hepatic tissue has blood supply from both the PV and the HA, a dual-blood input function (DBIF) that takes into consideration the tracer concentration in both vessels is believed to produce more reasonable results [10, 11]. The arterial input function can either be directly obtained by arterial blood sampling [5] or be derived from the left ventricle or aortic regions in dynamic PET images [12–14].Whereas the portal vein input function was usually estimated by using the convolution models of arterial input function with population-based parameters, or with individuation-based parameters determined together with kinetic modeling [10, 15–17]

Despite the previously reported studies, there is still no well-established ^18^F-FDG kinetic model in liver. The objective of the present study is to identify a simple and solid model for ^18^F-FDG kinetics in liver. A reversible two-tissue compartment model was used in this study. Image-derived input functions from both the HA and the PV were proposed, with an optimization derived blood supply fraction parameter [18] to describe the contribution of the HA to the blood input function. The efficacy of the presented liver kinetic modeling was evaluated by comparing the glucose metabolic characterization between malignant lesions and the healthy tissue, as well as between HCC and ICC. Kinetic parameters derived from time-activity curves (TACs) measured with different SUVs were also compared to identify the effect of SUV measurements on the kinetic modeling.

## Methods

### Patient selection

This study was approved by the Ethics Committee at Peking Union Medical College Hospital and all patients signed informed consent. 24 patients (20 male and 4 female, 36–74 years of age) with advanced HCC or ICC were recruited. These patients had received treatments including mixed anti-tumor drugs, local radiotherapy, transarterial chemoembolization (TACE), radiofrequency ablation (RFA) or immune checkpoint inhibitors (ICI). The patients were further divided into HCC group (9 patients, all male, 38–73 years of age) and ICC group (15 patients, 11 male and 4 female, 36–74 years of age) according to the prior surgical pathology.

### ^***18***^***F-FDG PET/CT scan***

PET/CT scans were performed on a PoleStar m660 PET/CT scanner (SinoUnion Healthcare, Beijing, China) at Peking Union Medical College Hospital (PUMCH) [19]. CT transmission scans (120 kV, 160 effective mA) were conducted first for attenuation correction and image fusion. The dynamic PET studies were performed over the liver region right after intravenous administration of 3.70—5.55 MBq/kg (0.10—0.15 mCi/kg) ^18^F-FDG and lasted for 60 min. A 50-frame sampling protocol consisting of 6 frames of 5 s, 3 frames of 10 s, 6 frames of 20 s, 14 frames of 30 s, 10 frames of 60 s, 5 frames of 120 s and 6 frames of 300 s was used. The interval between frames was gradually extended as the radioactivity decreased during the PET scan. Dynamic PET images were then reconstructed using ordered subset expectation maximization (OSEM) algorithm with 2 iterations and 10 subsets.

### Image analysis

Delineation of volumes of interest (VOIs) was done on a MIM workstation (MIM Software Inc, Cleveland, OH, USA). FDG avid tumor lesions with SUV more than 1.5-fold greater than the background uptake of normal liver tissues were selected. The VOIs were drawn over tumor lesions on the dynamic PET images of each patient. For tumor lesions, a fixed threshold of 40% SUVmax method was used, with manually adjustment slice by slice. For comparison, a reference VOI using a 2-3 cm diameter sphere was also delineated on the normal liver tissues for each patient. In addition, the VOIs of descending aorta and PV were also manually drawn slice by slice to obtain arterial and venous input function, respectively. The corresponding lesion volume was measured, and TACs consisting of SUVmax as well as SUVmean extracted from each frame were respectively generated.

**Fig. 1:**
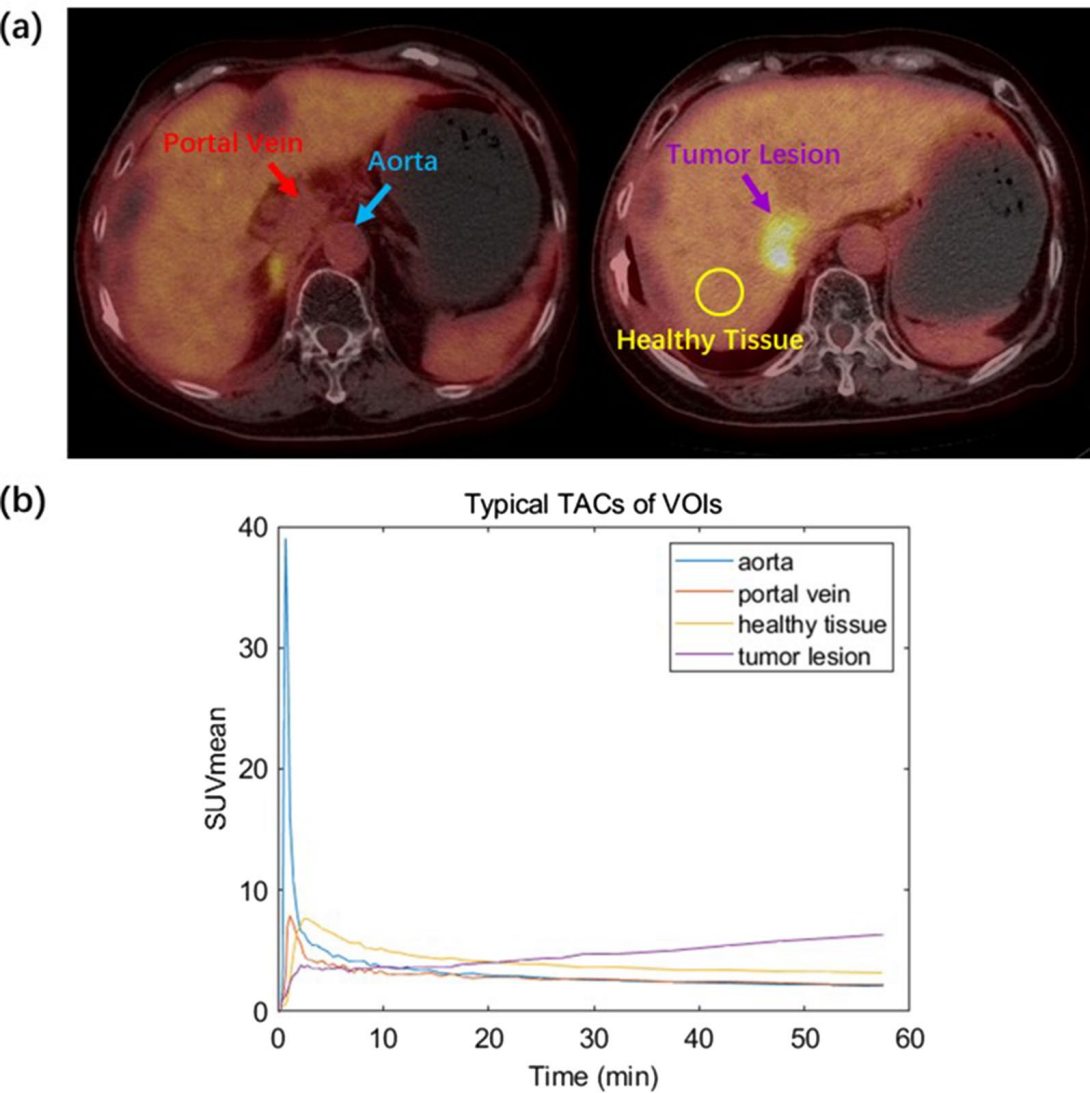
**a** Representative transaxial PET/CT images of a 73-year-old female patient with ICC. Arrows display the VOIs of aorta, PV, healthy tissue and tumor lesion, **b** typical TACs of the corresponding delineated VOIs

Figure 1a shows the transaxial ^18^F-FDG PET/CT in a patient with ICC where VOIs of the aorta, the PV, the healthy tissue and the tumor lesion were delineated. The corresponding measured TACs of this patient are plotted in Fig. 1b.

### Kinetic modeling

**Fig. 2:**
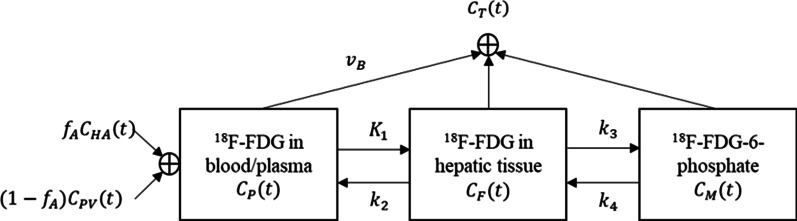
Two-tissue compartment model with dual-blood input function (DBIF)

A two-tissue compartment model with dual blood input functions was used in this study to describe the ^18^F-FDG kinetics in the liver. The kinetic model was implemented in-house using MATLAB (MathWorks, Natick, MA, USA). As shown in Fig. 2, ${C}_{HA}\left(t\right)$ represents the measured ^18^F-FDG concentration in the HA and ${C}_{PV}\left(t\right)$ in the PV. The parameter ${f}_{A}$ refers to the fraction of the HA contributing to the liver blood inflow. Therefore, the ^18^F-FDG concentration in the dual blood supply as well as in the plasma compartment ${C}_{p}\left(t\right)$, can be described as follows:

$${C}_{P}\left(t\right)=\left(1-{f}_{A}\right){C}_{PV}\left(t\right)+{f}_{A}{C}_{HA}\left(t\right)$$

where ${C}_{f}\left(t\right)$ and ${C}_{m}\left(t\right)$ denotes the free-state ^18^F-FDG concentration and the metabolized ^18^F-FDG 6-phosphate concentration in the hepatic tissue compartment respectively.

Kinetic rate constants ${K}_{1}$(ml/min/ml) represents the ^18^F-FDG delivery rate from blood to the hepatic tissue and ${k}_{2}$(1/min) represents the clearance rate back to the blood. ${k}_{3}$(1/min) is associated with the phosphorylation rate of ^18^F-FDG into ^18^F-FDG 6-phosphate by hexokinase and ${k}_{4}$(1/min) with the dephosphorylation rate by phosphatase.

${C}_{T}\left(t\right)$ is the output function of the kinetic model and it is the collective concentration including the blood capillary and hepatic tissue compartments, where ${v}_{B}$ is blood volume fraction representing the partial volume effect caused by capillaries:

$${C}_{T}\left(t\right)={v}_{B}\times {C}_{P}\left(t\right)+{C}_{F}\left(t\right)+{C}_{M}\left(t\right)$$

In this study, kinetic parameters ${K}_{1}-{k}_{4}$, hepatic arterial blood supplying fraction ${f}_{A}$ and blood volume fraction ${v}_{B}$ were unknown constant parameters and denoted as a vector, $\theta =[{K}_{1}, {k}_{2},{k}_{3}, {k}_{4},{f}_{A}, {v}_{B}]$.

### Estimation of kinetic parameters

Using the nonlinear least square estimation (NLS), the above unknown constant parameters were estimated by iteratively fitting the output function ${C}_{T}\left(t\right)$ with the TAC ${C}_{meas}\left(t\right)$ measured by PET:

$$\begin{aligned} \hat{\theta } & = arg\mathop {\min }\limits_{\theta } WRSS\left( \theta \right) \\ WRSS\left( \theta \right) & = \mathop \sum \limits_{i = 1}^{N} w_{i} \left[ {C_{meas} \left( {t_{i} } \right) - C_{T} \left( {t_{i} ;\theta } \right)} \right]^{2} \\ \end{aligned}$$

where $N$ is the number of frames, and a uniform weighting factor ${w}_{i}=1/N$, was used for each frame [17]. The weighted residual sum of squares (WRSS) is iteratively updated till it reaches the smallest, indicating the best curve fitting between ${C}_{T}\left(t\right)$ and ${C}_{meas}\left(t\right)$. The optimization problem was solved by Trust-region Algorithm and implemented using MATLAB version 9.5, R2018b (MathWorks, Natick, MA, USA). The initial parameters for iterations were set as ${K}_{1}$=1.0, ${k}_{2}$=1.0, ${k}_{3}$=0.01, ${k}_{4}$=0.01, ${v}_{B}$=0.01 and ${f}_{A}$=0.25 based on the population empirical values. For each VOI, a specific set of kinetic parameters ${K}_{1}-{k}_{4}$, ${f}_{A}$ and ${v}_{B}$ were estimated, and the combined metabolic rate constant ${K}_{i}={K}_{1}\times {k}_{3}/\left({k}_{2}+{k}_{3}\right)$ was also calculated.

### Comparison of SUV measurements

In order to evaluate the effect of different SUV measurements on the kinetic analysis, both the SUVmean and SUVmax measurements were used to respectively generate the TACs for reference and tumor lesion VOIs, and the two kinds of TACs went through the aforementioned kinetic analysis and results were compared. It is worthwhile to note that for the blood input functions, however, SUVmean measured over the descending aorta and PV VOIs were used to calculate ${C}_{HA}\left(t\right)$ and ${C}_{PV}\left(t\right)$ respectively, assuming a homogeneous tracer distribution in the blood flow.

### Statistical analysis

The fitting outcomes of the two kinds of TACs were compared using the corrected Akaike Information Criterion (AICc), due to the limited number of time frames. A smaller AICc value indicates a better curve fitting [20].

### Clinical data evaluation

The student’s t-test was used to assess the significance of difference in FDG kinetic parameters between the tumor lesions and the normal tissues (reference VOI), as well as in the lesions between HCC group and ICC group. The lesion volume and SUVmax extracted from the last 5 min of dynamic images were also reported. Quantitative data were expressed as mean ± standard deviation (SD). All statistical analysis were performed using SPSS 23.0 software (IBM SPSS, Chicago, IL, USA) and a *P*-value of < 0.05 was considered statistically significant.

## Results

### Clinical characteristics

For 10 out of the 24 patients enrolled in the study, more than one lesion was identified. In the HCC group, 9 patients (all males, 38–73 years of age) with 13 available lesions were studied. In the ICC group, 15 patients (11 males and 4 females, 36–74 years of age) with 25 available lesions were studied. The total number of lesions in all the patients was 38. For each patient, tumor lesions as well as a reference normal liver tissue VOI were delineated. Clinical characteristics are summarized in Tables 1 and 2.

**Table 1:** Patient and clinical characteristics of HCC group

Patient number	Gender	Age	Previous treatment	Liver cirrhosis	Lesion number	Lesion size (cm^3^)	Lesion SUVmax
1	M	59	Surgery, TACE, ICI	+	2	106.61	9.61
						3.95	9.47
2	M	64	RFA, ICI	+	1	1.78	4.69
3	M	67	Liver transplantation, radiotherapy, ICI	–	1	0.47	4.53
4	M	73	Surgery, TACE, RFA, ICI	–	1	213.25	9.42
5	M	46	Surgery, TACE, ICI	+	3	2.9	20.05
						1.63	20.96
						0.89	15.65
6	M	61	Surgery, TACE, RFA, ICI	+	1	4.4	7.39
7	M	71	Surgery, TACE, RFA, ICI	+	1	3.37	5.82
8	M	50	TACE, ICI	+	2	1.46	4.38
						2.30	3.31
9	M	38	TACE, radiotherapy, ICI	+	1	18.14	8.15
Mean ± SD		58.78 ± 11.85				27.78 ± 62.72	9.49 ± 5.87

TACE, transcatheter arterial chemoembolization; RFA, Radio Frequency Ablation; ICI, immune checkpoint inhibitor

**Table 2:** Patient and clinical characteristics of ICC group

Patient number	Gender	Age	Previous treatment	Lesion number	Lesion size (cm^3^)	Lesion SUVmax
1	M	36	RFA, ICI	3	0.74	9.24
					0.86	14.81
					0.51	17.43
2	M	42	Surgery	3	1.11	7.80
					2.40	9.94
					1.25	11.57
3	M	70	Surgery, radiotherapy, chemotherapy, gamma knife radiotherapy, ICI	1	2.95	5.10
4	M	61	ICI	1	106.97	12.05
5	M	47	Surgery, chemotherapy, RFA, ICI	3	1.82	4.49
					5.29	2.95
					82.40	5.26
6	F	73	Radiotherapy, chemotherapy, gamma knife radiotherapy, RFA, ICI	1	2.79	7.67
7	M	49	Surgery, chemotherapy, ICI	2	5.01	4.30
					15.85	5.01
8	M	52	Surgery, TACE, RFA, ICI	1	2.71	4.78
9	M	74	ICI	1	27.08	5.42
10	F	44	Surgery, chemotherapy, RFA, ICI	1	6.95	8.93
11	F	54	Surgery, chemotherapy	1	0.92	4.64
12	M	62	Surgery, chemotherapy, ICI	2	6.04	7.97
					5.22	6.83
13	M	55	TACE, chemotherapy, ICI	1	29.35	11.19
14	M	74	Surgery, TACE, ICI	2	8.72	4.22
					2.82	4.41
15	F	56	Surgery, chemotherapy, TACE, RFA	2	4.08	5.04
					4.80	5.97
Mean ± SD		56.60 ± 12.19			13.15 ± 25.89	7.48 ± 3.65

TACE = transcatheter arterial chemoembolization; RFA = Radio Frequency Ablation, ICI = immune checkpoint inhibitor

### TAC curve fitting

**Fig. 3:**
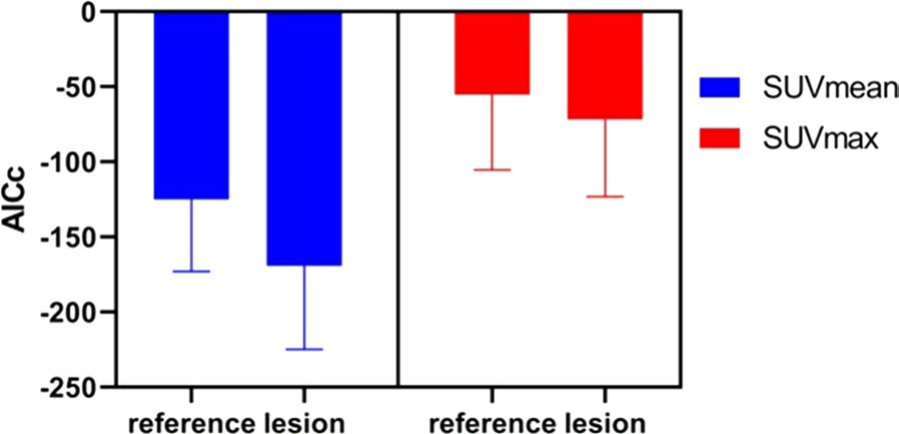
AICc values (mean ± SD) of TACs measured by SUVmax and SUVmean

**Fig. 4:**
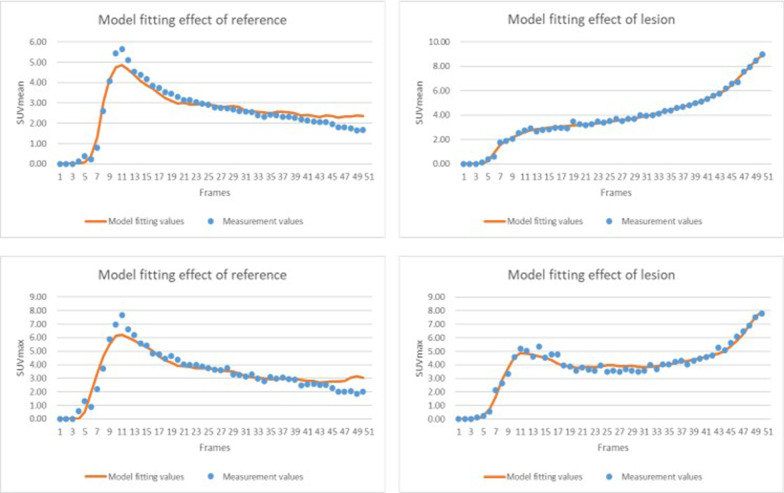
Example Curve fitting results of the TAC measured by SUVmax and SUVmean

Figure 3 shows the mean and standard deviation of AICc values for both the tumor lesions and the reference tissue, using SUVmax and SUVmean measurements respectively. In general, the AICc scores using SUVmean measurement are better compared to those using SUVmax measurement, due to the overall much smoother TACs averaged over the VOI when calculating the SUVmean. However, the curve fitting results, as shown in Fig. 4, demonstrated similar good quality of fitting for both TACs using either SUVmean or SUVmax measurements.

### Comparison of kinetic parameters

**Fig. 5:**
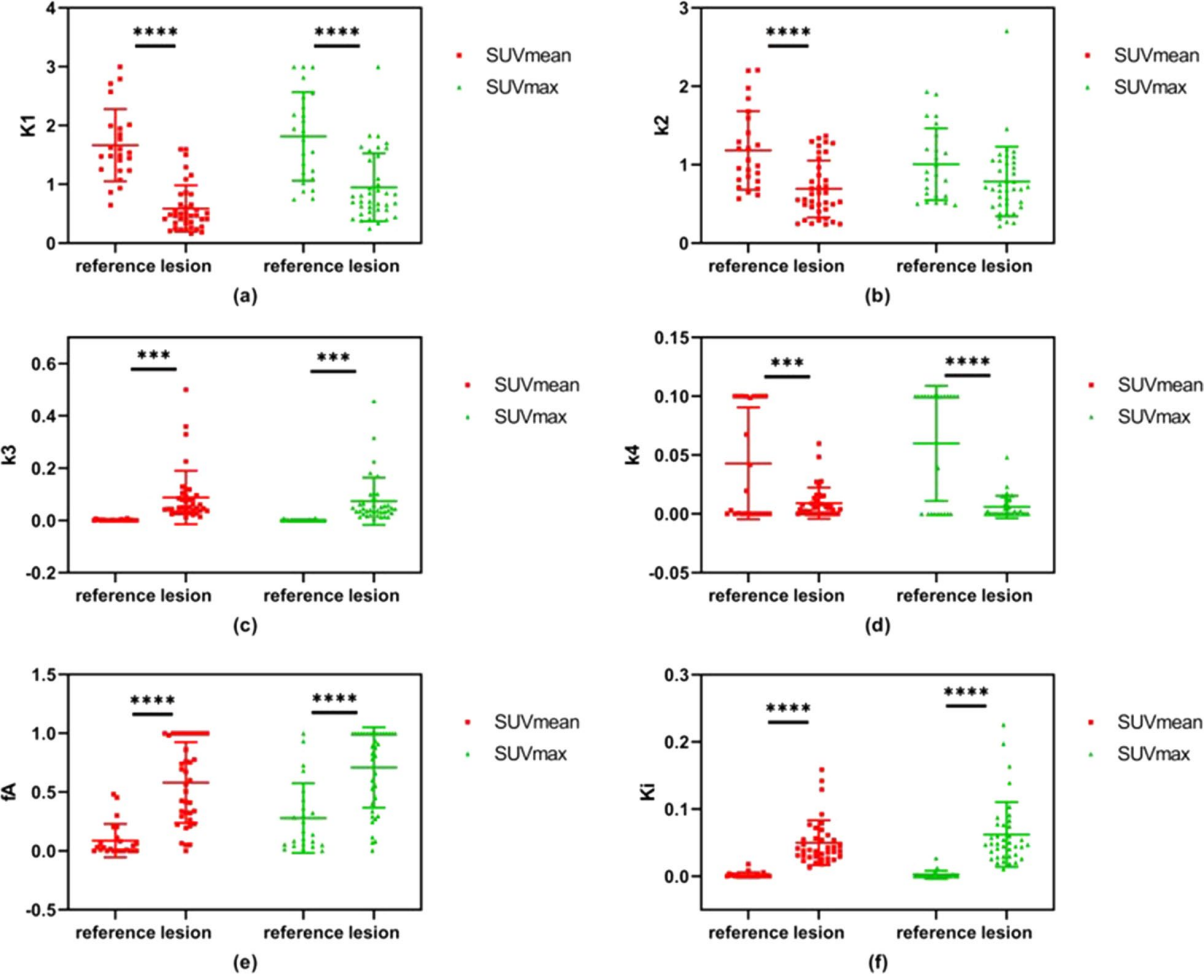
Kinetic parameters estimated from TACs of SUVmax and SUVmean, comparing healthy tissue and tumor lesions

As all VOIs were classified into the reference tissue or the tumor lesion group, Table 3 lists the mean and standard deviation of the kinetic parameters ${K}_{1}-{k}_{4}$, blood supplying fraction ${f}_{A}$, and metabolic rate constant ${K}_{i}$ of the above two groups, derived from TACs of both the SUVmean and SUVmax measurements. The scatterplots with group mean and standard deviation of these kinetic parameters are further shown in Fig. 5.

**Table 3:** Mean and standard deviation of kinetic parameters estimated from TACs with SUVmean and SUVmax, comparing tumor lesions and reference tissues

Parameters	SUVmean			SUVmax		
	Reference	Lesion	*P* value	Reference	Lesion	*P* value
*K*_1_	1.66 ± 0.61	0.59 ± 0.39	< 0.0001****	1.81 ± 0.75	0.95 ± 0.58	< 0.0001****
*k*_2_	1.18 ± 0.50	0.69 ± 0.36	< 0.0001****	1.01 ± 0.46	0.79 ± 0.44	0.064
*k*_3_	0.001 ± 0.002	0.088 ± 0.102	0.0001****	0.001 ± 0.003	0.073 ± 0.090	0.0002***
*k*_4_	0.043 ± 0.048	0.009 ± 0.013	0.0001****	0.060 ± 0.049	0.006 ± 0.010	< 0.0001****
*f*_*A*_	0.09 ± 0.14	0.58 ± 0.34	< 0.0001****	0.28 ± 0.30	0.71 ± 0.34	< 0.0001****
*ν*_*B*_	0.005 ± 0.015	0.016 ± 0.021	0.029	0.011 ± 0.020	0.025 ± 0.023	0.021
*K*_*i*_	0.002 ± 0.004	0.050 ± 0.033	< 0.0001****	0.002 ± 0.006	0.062 ± 0.048	< 0.0001****

****, ***, **, and * denote statistical significance at the 0.1%, 1%, 5%, and 10% levels, respectively

As shown in Table 3 and Fig. 5, kinetic parameters derived using SUVmean and SUVmax measurements respectively exhibit similar effectiveness in differentiating the reference tissues from tumor lesions. For reference tissues, the ${K}_{i}$ was 0.002 ± 0.004 and 0.002 ± 0.006 with the SUVmean and SUVmax measurements respectively; for tumor lesions, however, the ${K}_{i}$ was 0.050 ± 0.033 and 0.062 ± 0.048 with the SUVmean and SUVmax measurements respectively, indicating a much higher metabolic rate in lesions. The HA blood supply fraction ${f}_{A}$ was 0.09 ± 0.14 and 0.28 ± 0.30 for the reference tissues, with the SUVmean and SUVmax measurements respectively; while for tumor lesions, the ${f}_{A}$ was estimated to be 0.58 ± 0.34 and 0.71 ± 0.34 with the SUVmean and SUVmax measurements respectively, indicating a much higher portion of HA blood supply in lesions compared with normal liver tissue. The student’s t-test shows significant difference when comparing the results from the reference tissues and tumor lesions, using either SUVmean or SUVmax measurements, except for one case when comparing the ${k}_{2}$ between the reference tissues and tumor lesions obtained with the SUVmax.

**Fig. 6:**
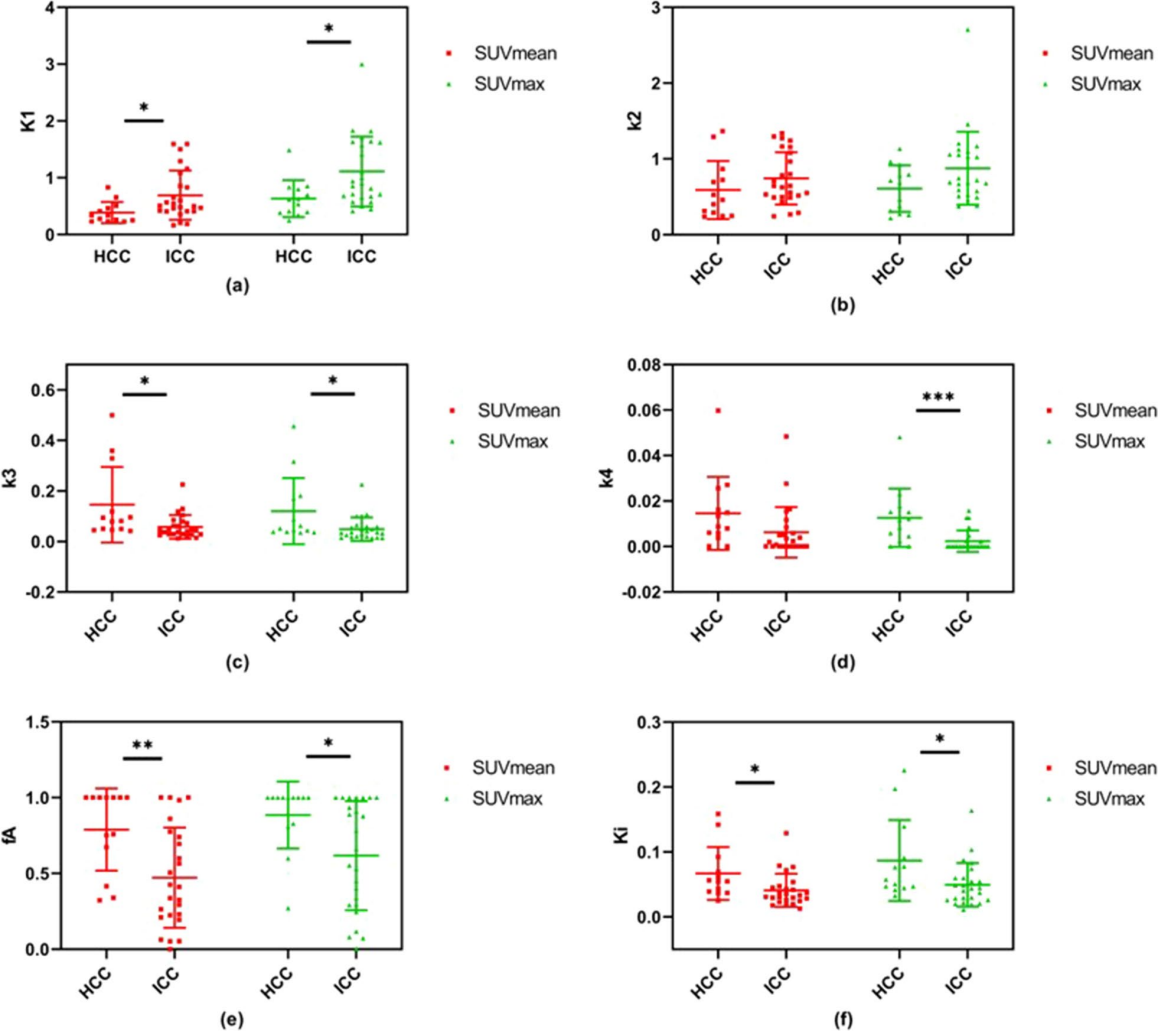
Kinetic parameters estimated from TACs of SUVmax and SUVmean, comparing HCC and ICC

To further investigate the relation of kinetic analysis and pathology, the tumor lesion VOIs were divided into HCC and ICC group. The kinetic parameters derived from TACs with both SUVmean and SUVmax measurements are provided in the mean ± SD format and listed in Table 4. And Fig. 6 shows the scatterplots with group mean and standard deviation of these kinetic parameters.

**Table 4:** Mean and standard deviation of kinetic parameters estimated from TACs of SUVmax, and SUVmean, comparing HCC and ICC

Parameters	SUVmean			SUVmax		
	HCC	ICC	*P* value	HCC	ICC	*P* value
*K*_1_	0.39 ± 0.19	0.69 ± 0.43	0.0217*	0.63 ± 0.32	1.11 ± 0.62	0.0135*
*k*_2_	0.59 ± 0.38	0.74 ± 0.35	0.2197	0.61 ± 0.31	0.88 ± 0.48	0.0764
*k*_3_	0.145 ± 0.150	0.058 ± 0.047	0.0103*	0.120 ± 0.131	0.049 ± 0.046	0.0185*
*k*_4_	0.015 ± 0.016	0.006 ± 0.011	0.069	0.013 ± 0.013	0.002 ± 0.005	0.0009***
*f*_*A*_	0.79 ± 0.27	0.47 ± 0.33	0.0052**	0.89 ± 0.22	0.62 ± 0.36	0.0194*
*ν*_*B*_	0.015 ± 0.017	0.017 ± 0.023	0.74	0.028 ± 0.023	0.023 ± 0.023	0.57
*K*_*i*_	0.067 ± 0.041	0.041 ± 0.025	0.0209*	0.087 ± 0.062	0.049 ± 0.033	0.0207*

****, ***, **, and * denote statistical significance at the 0.1%, 1%, 5%, and 10% levels, respectively

In general, kinetic parameters derived using SUVmean and SUVmax measurements respectively perform similar in terms of HCC and ICC differentiation. The ${K}_{i}$ for HCC was 0.067 ± 0.041 and 0.087 ± 0.062 with the SUVmean and SUVmax measurements respectively; while for ICC, the ${K}_{i}$ was 0.041 ± 0.025 and 0.049 ± 0.033 with the SUVmean and SUVmax measurements respectively, indicating a decreased metabolic rate in ICC compared to HCC lesions. The HA blood supply fraction also shows a decrease, with ${f}_{A}$ of 0.79 ± 0.27 and 0.89 ± 0.22 for HCC, with the SUVmean and SUVmax measurements respectively; and 0.47 ± 0.33 and 0.62 ± 0.36 for ICC, with the SUVmean and SUVmax measurements respectively. The student’s t-test shows significant difference in ${K}_{1}$, ${k}_{3}$, ${f}_{A}$ and ${K}_{i}$ between HCC and ICC, using either SUVmean or SUVmax measurements. The ${k}_{4}$ derived using SUVmax also shows significant difference of almost a magnitude between HCC and ICC, while for the same parameter derived using SUVmean, no significant difference was observed between HCC and ICC.

## Discussion

Kinetic modeling allows quantification of biochemical process of a tracer in a certain region from measured tissue TACs and input functions. In this study, a reversible (${k}_{4}\ge 0$) two-tissue compartment model with DBIF, which takes into consideration the blood supply from both the HA and the PV, was used for accurate kinetic modeling of liver dynamic ^18^F-FDG PET imaging. While in previous studies [14–16], the PV input function was estimated by using the convolution models of arterial input function, the PV input function in the presented study was directly measured as the mean values over the VOIs on the PV. And similar to that proposed by Wang et al. [15], the contribution of the HA to the blood input function, denoted as ${f}_{A}$, was optimization-derived in the process of model fitting, instead of using a population-based fixed parameter [14]. Evaluations of the kinetic model showed significant differences (in most cases $p<0.0001$) in kinetic parameters ${K}_{1}-{k}_{4}$, blood supplying fraction ${f}_{A}$, and metabolic rate constant ${K}_{i}$ between malignant lesions and healthy liver tissue.

It is known that the blood supply to healthy hepatic tissue and malignant lesions is different. The healthy hepatic tissue is mainly supplied by the PV, which carries 70%-80% of overall inflow. While the malignant lesions such as HCC is a hypervascular tumor mainly supplied by the HA, so the proportion of arterial supply is significant higher in tumor lesions than in the healthy tissue. The results in this study are in consistence with these previous findings, with a significant increase of ${f}_{A}$ in tumor lesions compared with the healthy liver tissue [21, 22].

^18^F-FDG is transported across the cell membrane by glucose transporters (Gluts), and phosphorylated by hexokinase into ^18^F-FDG-6-phosphate, which cannot be metabolized and trapped in the cells. Meanwhile, the dephosphorylation of ^18^F-FDG-6-phosphate back into ^18^F-FDG by phosphatase occurs at the same time. High levels of glucose-6-phosphatase are found in normal liver, leading to dephosphorylation of ^18^F-FDG, which subsequently no longer accumulates in cells and redistributes back into the circulation. The results in this study showed that compared with healthy liver tissue, the phosphorylation rate ${k}_{3}$ and metabolic rate constant ${K}_{i}$ exhibit significant increase in tumor lesions. This is in accordance with previous studies that demonstrated increased hexokinase activity in malignant tissue using immunohistochemistry [22–24].In addition, ${k}_{4}$ of healthy liver were significantly higher than that of tumor lesions, reflecting higher dephosphorylation activity in healthy liver tissues. In our results, the ${K}_{1}$ shows significant decrease in tumor lesions compared with healthy liver, although previous study showed increased Gluts in malignance [23]. The decreased K1 observed in this study may be the result of a significant increase in the HA supply to the lesions, because with a much higher portion of HA supply, adequate blood perfusion and oxygen supply to the hepatic tissues can still be achieved at a decreased transport rate. But it is also worthwhile to note that the antiangiogenic effect caused by the treatments that the patients received prior to our study may also have contributions. In addition, 7 out of the 9 HCC patients in this study reported a history of liver cirrhosis. Liver cirrhosis is characterized by nodular regeneration of liver tissue with the destruction of the lobular and vascular architecture. Decreased glucose metabolism and decreased expression level of GLUT-4 was reported in liver cirrhosis patients [28, 29]. This may also contribute to the decreased K1 observed in this study. Nevertheless, the overall differences observed in our study is consistent with previous literature reports [23, 25, 26], and the results indicate that the presented DBIF model can be used to describe the ^18^F-FDG kinetics in both the malignant and healthy liver regions.

The derived kinetic parameters were further analyzed to distinguish HCC from ICC in our study. It was reported that pathological overlap can be observed between HCC and ICC, which made the differentiation difficult in practice [27]. Our results showed that using the presented DBIF model, HCC and ICC can be further differentiated with ${K}_{1}$, ${k}_{3}$, ${f}_{A}$ and ${K}_{i}$ ($p<0.05$). ${K}_{1}$ of the HCC are smaller than those of ICC, while ${k}_{3}$, ${f}_{A}$, and ${K}_{\mathrm{i}}$ are higher in HCC than in ICC. This indicates that ICC tumors accumulate ^18^F-FDG from blood to tissue faster, while HCC tumors convert ^18^F-FDG into its metabolite in the tissue more actively with higher blood supply from HA. This is in accordance with previous studies using transcriptomic analysis which revealed up-regulation of GLUT-1 in ICC relative to HCC, and down-regulation of lipid pathways, suggesting metabolic differences between HCC and ICC [28]. Despite the observed differences in kinetic parameters between HCC and ICC, it is also worthwhile to note that the patients’ prior treatments may have impact on the FDG dynamic processes in these lesions.

**Fig. 7:**
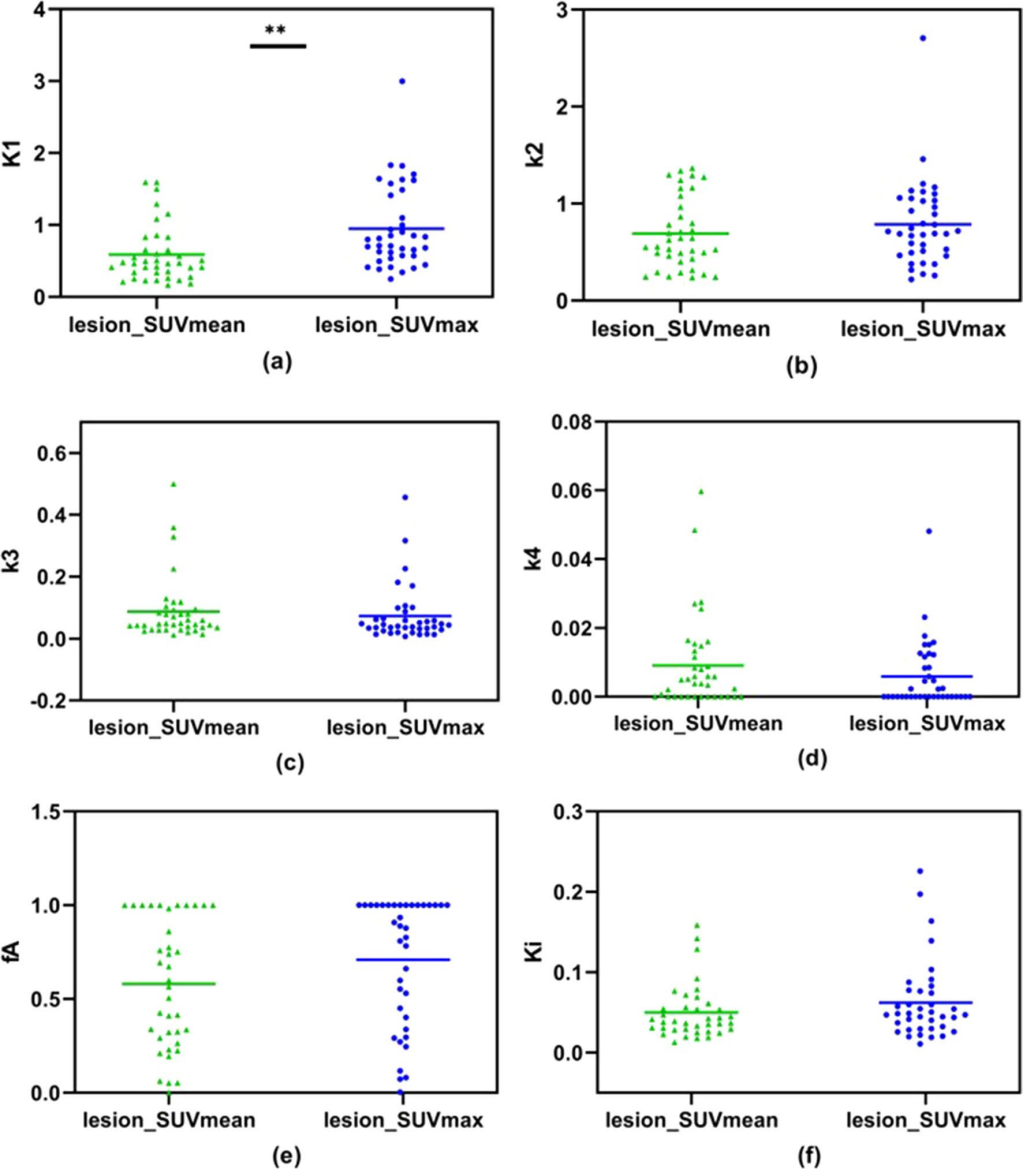
Comparison of kinetic parameters estimated from TACs using SUVmax and SUVmean respectively

The presented study also investigated the effect of SUV measurements on derived kinetic parameters with the proposed DBIF kinetic model. Despite the fact that SUVmax is most commonly used for lesion diagnosis in static PET imaging and almost no interobserver variability was found for the measurement [29], it is less commonly used in dynamic PET imaging for TACs extraction due to its vulnerability to noise. However, it is well known that tumor lesions had heterogeneous metabolism, meaning uneven uptake of ^18^F-FDG in the lesions, and SUVmax is more preferred to reflect the heterogeneity. We compared the kinetic parameters derived with SUVmean and SUVmax respectively. In general, with the proposed DBIF kinetic model, SUVmax performs similar to SUVmean in differentiating malignant and healthy liver tissue as well as in differentiating HCC and ICC, as shown in the results. No significant difference was found for kinetic parameters except for ${K}_{1}$ using either SUVmean or SUVmax, as shown in Fig. 7. These findings suggest the robustness of the kinetic analysis to the SUV measurements using the proposed DBIF kinetic model. A potential perspective is the proposed model to be implemented on a voxel-by-voxel manner to derive parametric images [30–32] which will allow further study of liver heterogeneity. In this case, the VOI will become a voxel and the SUV on each individual voxel will be used. Our comparison between SUV measurements suggests that the parametric images are predictable when extending the proposed DBIF kinetic model to the voxel level.

The comparison between results derived using SUVmean and SUVmax respectively, however, also reveals some interesting findings. For healthy liver, the HA blood supply fraction ${f}_{A}$ was estimated to be 0.28 ± 0.30 using SUVmax, indicating that the HA accounts for about 28% blood supply to healthy liver, which is consistent with clinical experience value of 20–30% [11]. While using SUVmean, ${f}_{A}$ was estimated to be 0.09 ± 0.14, which is considerably lower than expectation. Moreover, the estimated ${f}_{A}$ for tumor lesions using SUVmean is 0.58 ± 0.34 and is comparably less than the 0.71 ± 0.34 by using SUVmax. This suggests that in general, using SUVmean tends to underestimate the HA blood supply while using SUVmax tends to provide a more reasonable estimation of the blood supplies to liver tissues. Also, when comparing the results between the HCC and ICC, it is interesting to note that using SUVmax, a significant reduction ($p=0.0009$) in ${k}_{4}$ was found in ICC compared with HCC, suggesting a significantly decreased dephosphorylation in ICC compared with HCC.

There are several limitations in our study. Firstly, all the patients have been treated with different therapies prior to the dynamic FDG scan. The inhomogeneity of the patients included in the study and the different treatment protocols used prior to our study may have an impact on both FDG-avidity and the blood input functions. A more controlled study is needed to further validate the results obtained in this study. Secondly, the dynamic PET was performed allowing patients to breathe freely, which may lead to mismatch between CT and PET images or slight position change of lesions. Motion correction or respiratory-gated PET could be performed to increase the accuracy of lesion delineation. Moreover, the VOIs size of the PV in some cases were small. And we did not consider the influence of partial volume effect in those VOIs. In addition, we have consulted a lot of literature on whether the upper limit of K value should be set [17, 33–35]. From the perspective of mathematical formula deduction, the value of K can exceed 1, but some literature mentioned that the value representing the rate should be artificially set at an upper limit of 1 in consideration of the physiological implications [36]. The feasibility and rationality of this view need further study and discussion. Lastly, the data of one-hour dynamic FDG imaging was difficult to obtain because of poor patients compliance, which made it difficult to recruit patients. The limited number of patients could affect the strength of our results. Further investigations with a larger number of patients are required.

## Conclusion

In this study, we proposed to use a reversible two-tissue compartment model with DBIF and optimization-derived HA blood supply fraction for the accurate kinetic modeling of liver dynamic ^18^F-FDG PET imaging. The kinetic model was found to effectively distinguish malignant lesions and healthy liver tissue, and it can also be used to further differentiate between HCC and ICC lesions. Both SUVmean and SUVmax were used to derive the kinetic parameters, and results suggest comparable effectiveness in performance between the two SUV measurements.

## Data Availability

The datasets generated and analyzed during the current study are not publicly available due to the security of data but are available from the corresponding author on reasonable request.

## Abbreviations

HCC: Hepatocellular carcinoma
ICC: Intrahepatic cholangiocarcinoma
^18^F-FDG PET: ^18^F-fluorodeoxyglucose positron emission tomography
SUVs: Standard uptake values
SBIF: Single-blood input function
DBIF: Dual-blood input function
TACs: Time-activity curves
OSEM: Ordered subset expectation maximization
NLS: Nonlinear least square estimation
WRSS: Weighted residual sum of squares
AICc: Corrected Akaike Information Criterion
SD: Standard deviation
TACE: Transcatheter arterial chemoembolization
RFA: Radio frequency ablation
ICI: Immune checkpoint inhibitor
